# Electroforming in VO_2_ Switch: Phase Transformation and Electromigration Phenomena

**DOI:** 10.1021/acsnano.5c19805

**Published:** 2026-04-01

**Authors:** Vanessa Conti, Cyrille Masserey, Victor Boureau, Anna Varini, Igor Stolichnov, Adrian Mihai Ionescu

**Affiliations:** † Nanoelectronic Devices Laboratory (NanoLab), 27218 Ecole Polytechnique Fédérale de Lausanne (EPFL), Lausanne 1015, Switzerland; ‡ Centre Interdisciplinaire de Microscopie Electronique (CIME), Ecole Polytechnique Fédérale de Lausanne (EPFL), Lausanne 1015, Switzerland

**Keywords:** vanadium oxide, mott insulators, complex oxides, electroforming, electromigration, memristors

## Abstract

Transition metal oxides provide a versatile platform for memristive devices, with vanadium oxide compounds attracting particular attention due to their sharp and ultrafast insulator-to-metal transition. However, their practical implementation is often constrained by structural and electrical modifications induced by high electric fields, commonly referred to as electroforming. In this work, we investigate the electroforming process in VO_2_ thin films deposited by atomic layer deposition and pulsed laser deposition, analyzing their microstructure and functional characteristics before and after electrical actuation. Detailed examination of the VO_2_ channel morphology and cross sections reveals pronounced phase transformations and electromigration phenomena associated with the switching process. Scanning transmission electron microscopy analysis reveals substantial structural reconfiguration, formation of new phases, and oxygen redistribution under electrical stress. These transformations result in noticeable modifications of key device parameters, including a median decrease of up to 72% in threshold voltage and 95% in channel resistance, ultimately reducing the ON/OFF ratio up to a factor of 160 in our experiments. At the same time, controlled electrical conditioning provides a pathway to exploit electroforming for reducing the stochasticity of switching events and enhancing fatigue resistance over >10^3^ voltage cycles. Overall, this study elucidates the interplay between structural evolution and electrical functionality in VO_2_-memristors, providing insights for enhancing their stability, reproducibility, and long-term reliability of VO_2_ switches.

## Graphical abstract

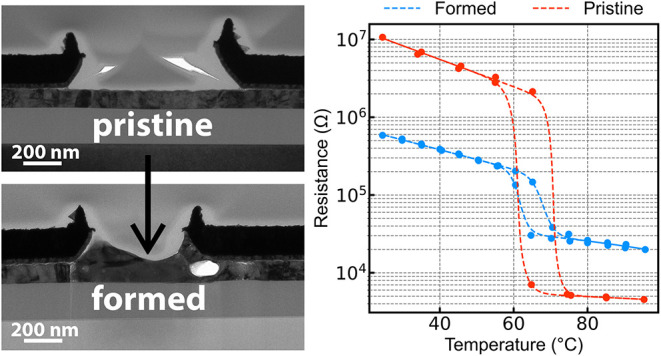

In recent years, neuromorphic systems have gained exponentially growing attention from the perspective of a highly efficient computation paradigm, enabling a reduction in terms of energy cost and area occupation. Inspired by brain operation, communication within these architectures is carried by circuit elements emulating the role of neurons and synapses.^1^ In this context, memristors are particularly attractive due to their intrinsic ability to change their states in response to electrical stimuli. They rely on different mechanisms (e.g., ion migration, phase transition), all driven by the structural properties of the constituent material.^2^ Various transition metal oxides (TMO) have been studied as memristic components, owing to characteristics such as oxygen-vacancy-mediated ionic conduction (TiO_*x*_, TaO_*x*_, WO_*x*_, HfO_*x*_···), ferro- and antiferroelectricity (HfO_2_, ZrO_2_ compounds and alloys), and insulator-to-metal transition (V, Nb, and Ni oxides···).^3–7^ Notably, the V–O system represents an important class, as several compounds (V_2_O_3_, V_3_O_5_, VO_2_, V_2_O_5_···) exhibit an insulator-to-metal transition. Particularly, VO_2_ is one of the most explored candidates. Remarkably, prior to the transition, the VO_2_ crystal structure is in the monoclinic M1 phase (space group *P*2_1_/*c*), which is insulating and characterized by V atoms dimerized in chains. Upon transition, the structure transforms into the tetragonal rutile R phase (space group *P*4_2_/*mnm*), and the material becomes electrically conductive.^8^ For consistency with the literature, we refer to the VO_2_ phases using their conventional phase names together with their corresponding crystallographic space groups. Although the physical origin of the transition is still under debate and utterly complicated by the significative numbers of polymorphs,^9,10^ the huge change in resistivity up to 5 orders of magnitude after transition, its proximity to room temperature (68 °C in bulk crystals), and the incredible ultrafast response (up to fs^11^) made the material appealing as building block for neuromorphic systems.

Despite the great properties and flexibility given by the tunability of TMO phase and composition, they are subjected to electroforming effects, which may limit their implementation in functional memristors,^12,13^ as conductive paths are formed in the device channel under the combination of electric field and Joule heating. Although electroforming is required in certain memristors to enable switching between high- and low-resistance states, the phenomenon is inherently stochastic, with the main risks involving oxygen loss, formation of inclusions with different stoichiometry, and microstructure rearrangement, up to the complete failure of the device in operation. In V–O compounds, electroforming is not yet fully characterized,^14–17^ as the large number of species with similar oxygen-to-vanadium ratio, each potentially exhibiting multiple polymorphs, complicates the identification of different phases, resulting in an incomplete description of the process. Consequently, studies on polycrystalline VO_2_ often employ either thick films or thin films with a low insulator-to-metal resistance ratio to mitigate the impact of electroforming, while limiting film scalability and performance. In addition, although electroformed devices were reported to demonstrate interesting functional properties, the mechanism remains less explored, highlighting the need for further investigation into the reproducibility and characterization of the final formed device.^18–21^

In this work, we combine electrical characterization at the nanoscale and in two-terminal devices with advanced TEM techniques to investigate electroforming in VO_2_ as a starting point to fabricate memristors with predictable switching behavior. The consequences of electroforming on electrical performances in terms of insulator-to-metal parameters and stochasticity are addressed, with a link to the morphological characteristics of the pristine films. The role of structural elements during and after electroforming is illustrated, with insights into the resulting crystal phases. Finally, electromigration in VO_2_ is described as a consequence of defect formation during electroforming.

## Results and Discussion

### Electroforming at the Nanoscale

A 40 nm-thick film deposited with atomic layer deposition (ALD) was characterized with an AFM setup to study in situ the extent of structural damage after electrical actuation. The voltage is applied between the tip (grounded) and Cr/Au (10 nm/100 nm) contacts deposited on top of the surface, enabling emulation of a lateral device with flexible geometry constraints. In Figure 1a, topography images acquired before and after measuring the local *I*–*V* characteristics reveal surface modifications. In a pristine state, the roughness exceeds 5 nm, while upon the first voltage sweep, the surface rearranges, creating material thickening at the ground point, with smoother areas (<2 nm roughness) present in its vicinity. These smooth regions extend following the electric field lines toward the contact, and they show distinct conduction properties, being more resistive, thus suggesting recrystallization or amorphization. The presence of unaltered grains at the edge of these smooth regions (beyond the white dashed lines in the figure) indicates the existence of a preferential conduction path over the shortest one between the ground point and the contact. In Figure 1b, the same experiment is repeated by placing the tip on a grain directly contacting the metal line. In this configuration, the reduced number of available conduction paths and the narrower distribution of the field lines lead to a less severe alteration of the morphology in the surrounding grains.

**Figure 1:**
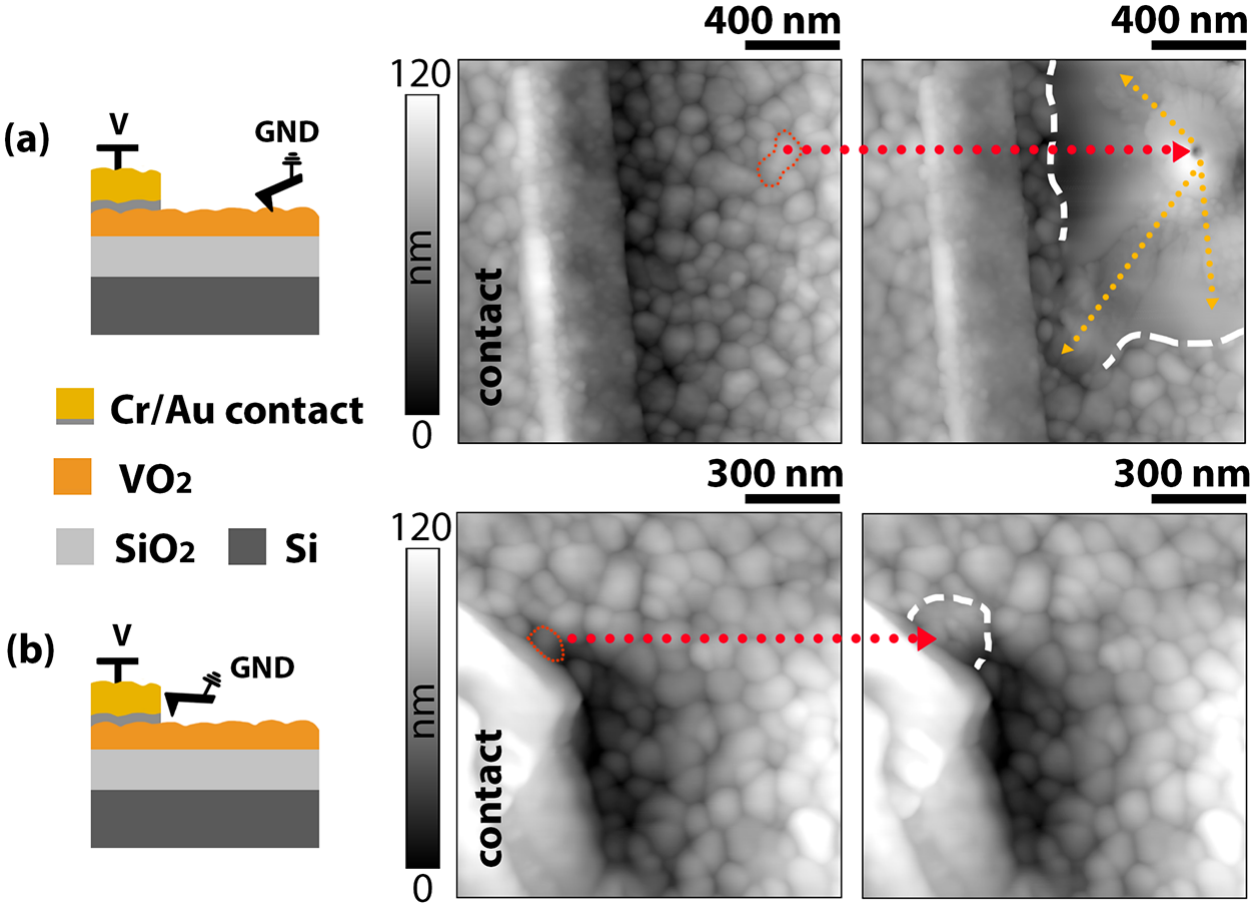
(a) Topography before and after actuation for a far placement of the tip with respect to the contact, as illustrated in the schematic. Electric field lines provoke widespread damage in several directions (yellow arrows). (b) Effect on topography for a close placement of the tip to the contact. Particularly, a grain directly in touch with the metal line is being investigated. For both cases, the red dotted lines indicate the grain where the tip is landed before actuation, while the white dashed lines delineate the region of visible surface modification after actuation.

In Figure 2a,b, the effect of electroforming on the I–V characteristics is reported for the scenarios in Figure 1. A reduction of the threshold voltage *V*_TH_ from insulating to metallic state is observed, and the magnitude of *V*_TH_ decrease depends on the tip position, as a further placement results in a larger reduction compared to the near-tip case, explained by a larger rearrangement of the surface. To understand the magnitude of the reduction, the experiment was repeated by measuring pristine *V*_TH_ and *V*_TH_ after actuation as an average of 25 measurements for multiple grains directly contacting the top electrode to minimize the number of possible conduction paths. As summarized by Figure 2c,d, a consistent reduction of the threshold voltage is observed independently of the grain area or the distance between the tip position and the contact, with an average relative decrease of about 50%.

**Figure 2:**
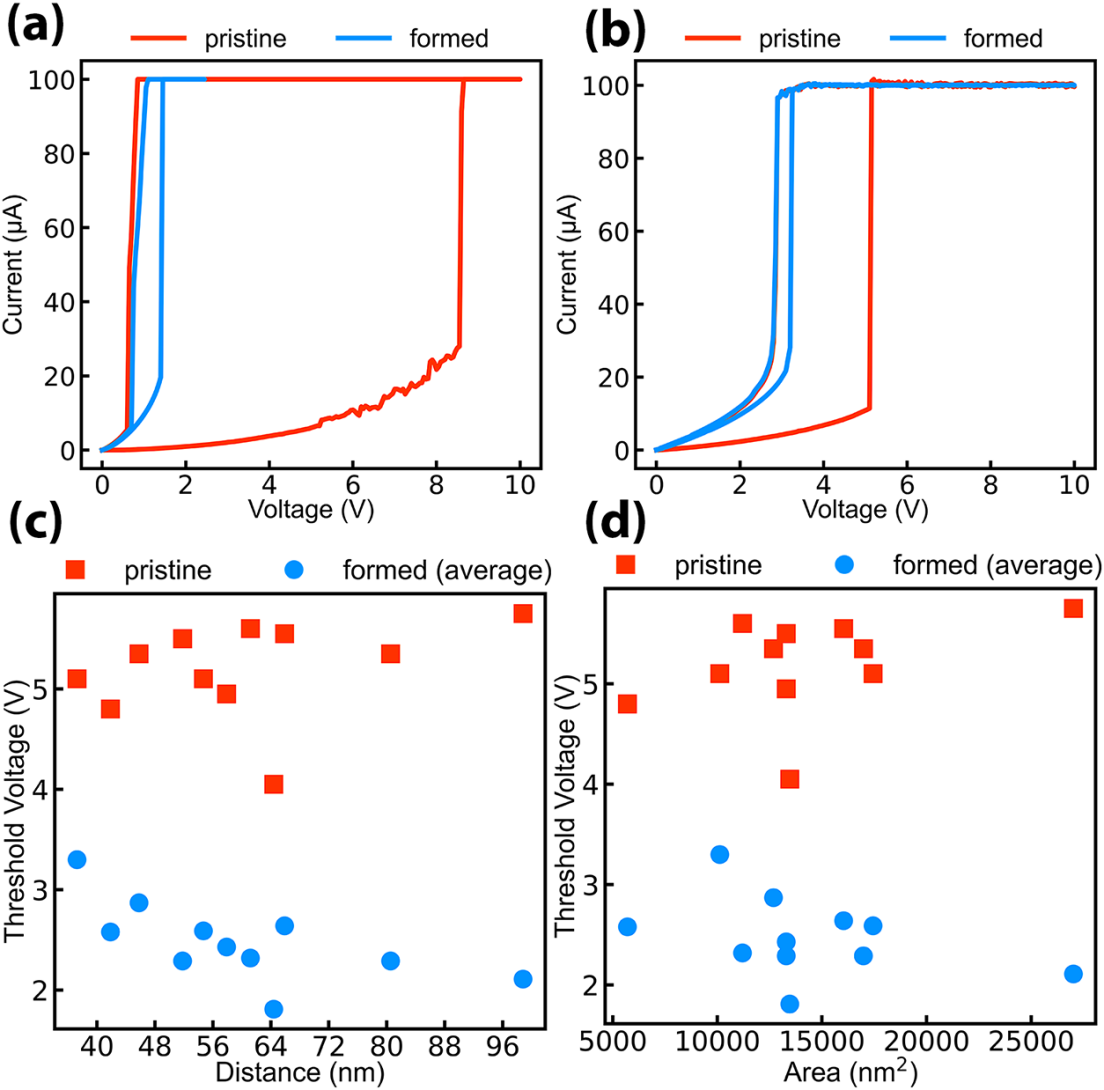
(a, b) *I*–*V* characteristics acquired for the points in Figure 1, respectively, for a far (a) and close placement of the tip (b). A larger *V*_TH_ reduction is observed for more aggressive surface modification. (c, d) *V*_TH_ difference between the first run (pristine state) and the average of the subsequent measurements for grains directly touching the contact. Reduction is insensible to the tip-contact distance (c) or the grain area (d).

We observe that the degree of electroforming is connected to the stochastic behavior of VO_2_. Figure 3a shows the progressive transformation of the surface after actuation with a low current compliance level (*I*_max_ = 30 μA) alongside the respective forward *I*–*V* sweeps (Figure 3b). As a consequence of the continuous forming process, the insulating resistance varies from run to run with a concomitant decrease of *V*_TH_. Figure 3c presents an example of 100 consecutive *I*–*V* curves acquired with a higher level of current compliance (*I*_max_ = 100 μA). In this case, the greater degree of electroforming following the first actuation minimizes the chance of further surface modifications, resulting in narrow distributions of both threshold voltage and current. The stable behavior of formed regions, compared with those where the process is incomplete, indicates that the origin of stochasticity in polycrystalline VO_2_ is primarily related to a direct alteration of the film characteristics during actuation rather than from the intrinsic switching probability of individual grains within a conduction path. In the situation depicted in Figure 3a, the area of interest is in direct contact with the metal line, excluding the possibility of multiple conduction paths during conductive filament formation. From an application perspective, this suggests that controlled and homogeneous electroforming can serve as an effective method to reduce the stochasticity of VO_2_ memristors.

**Figure 3:**
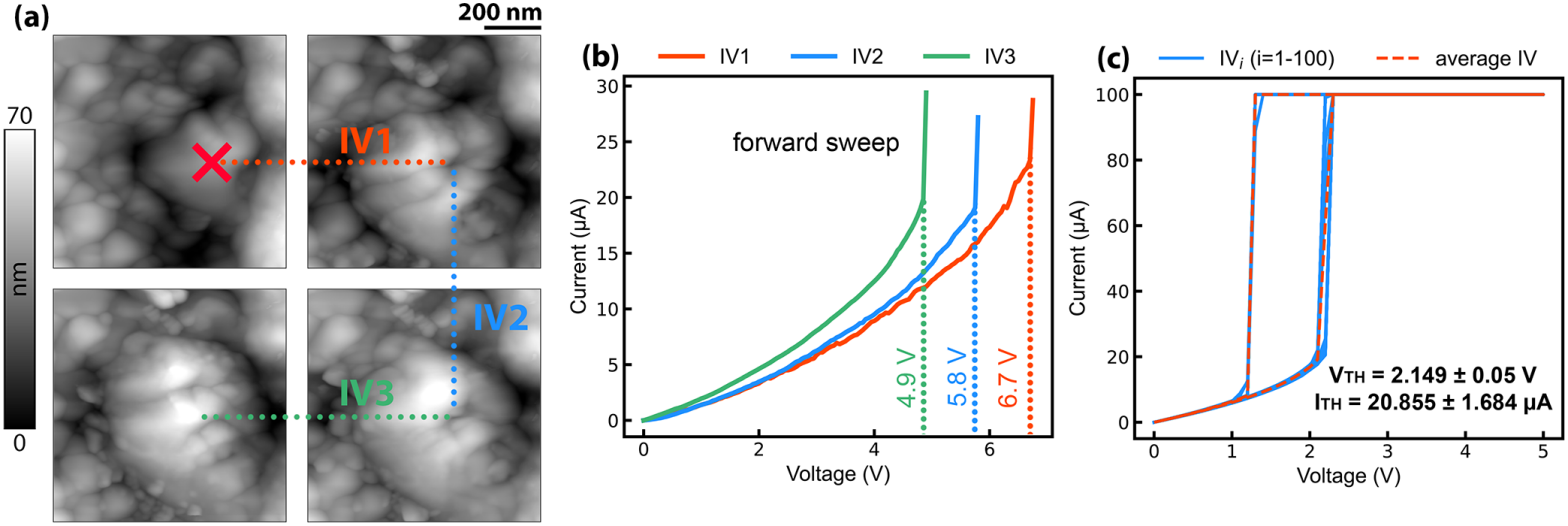
(a) Effect of partial electroforming on the topography of the same grain measured after several voltage sweeps IV1, IV2, and IV3. (b) Forward *I*–*V* characteristics IV1, IV2, and IV3 with the respective *V*_TH_. (c) Example of 100 consecutive *I*–*V* curves acquired on a heavily formed region. The average upper threshold parameters were obtained with a voltage step of 100 mV.

### Electroforming in Two-Terminal Devices

We systematically investigated electroforming in two-terminal devices with two distinct channel lengths (*L*) but the same width of 1 μm. Short-channel devices (*L* = 800 nm) were designed to uniformly electroform the channel by current preconditioning (Figure S1), reducing the risk of device breakage often observed for longer channels, and they were the sole type of device used for electrical characterization. Long-channel devices (*L* = 1.6 μm) were primarily fabricated to facilitate the acquisition of C-AFM maps and were also used for part of the structural investigation.

The forming procedure was conducted under controlled conditions: a first surface rearrangement is triggered by a current sweep to ensure that most of the channel undergoes modification, and then a voltage sweep is performed to register the *I*–*V* characteristic. Each measurement was repeated at least 35 times with a maximum voltage step of 50 mV to assess the threshold distributions after forming. During the formation process and often directly at the current-preconditioning step, some devices experienced catastrophic failure, typically manifested as an abrupt transition to an open-circuit state. The survivability rate (SR) is defined as the fraction of devices that remain functional after the forming procedure.

Three different samples were deposited with ALD and pulsed laser deposition (PLD) techniques: sample A (same as for the nanoscale investigation) and samples B (100 nm) and C (45 nm). The latter are deposited with PLD under different pressure and temperature conditions (see Methods). The films were characterized in detail in a previous work.^22^ In the following, only the characteristics relevant to the behavior of the devices will be discussed. Figure 4a illustrates the differences in their morphology. Sample A exhibits a homogeneous surface characterized by grains with a defined, rounded morphology. Sample B surface maintains high homogeneity, despite the presence of grains with a different shape, smaller and elongated at the points where nucleation occurs during deposition.^22^ Sample C surface is irregular, with the presence of small inclusions with various morphologies, attributed to a V–O phase with an increased oxygen content owned to the higher deposition pressure. The heterogeneous morphology impacts the sample’s behavior prior and after electroforming, as summarized by Figure 4b showing the insulating resistance (R_*I*_) before and following voltage actuation for multiple devices. The uniformity of the film microstructure can be directly assessed through the variability of pristine insulating resistance *R*_I_. Sample A exhibits the smallest dispersion, followed by sample B, while sample C shows a markedly larger variability. The effective channel length was measured for each short-channel device to evaluate the impact of fabrication variability on the R_I_ dispersion. Variations of ±80 nm (±10% of the nominal length) were observed, which are insufficient to account for the observed differences in *R*_I_. Furthermore, only a weak correlation (|*r*| < 0.3) was found between *R*_I_ and the effective channel length for all of the samples. After forming, a global reduction of about 1 order of magnitude in *R*_I_ is observed for samples A (−89% median variation m.v.) and B (−95% m.v.), whereas sample C shows a more marginal variation (−37% m.v.). The modest change observed in sample C relates to the magnitude of its resistance jump at the transition. Figure 4c reports *V*_TH_ before and after actuation. Regardless of the sample under investigation, *V*_TH_ in formed devices is generally smaller than its pristine value, with a m.v up to −72% observed in sample A. Using the measured effective channel lengths, the electric field at the first electroforming event was estimated to be 10.1, 7.06, and 4.93 MV/m for samples A, B, and C, respectively. These values, together with the DC measurement conditions, suggest Joule heating-dominated electrically driven switching.^23–25^ Under such conditions, a lower *V*_TH_ can result either from a reduced resistivity of the insulating phase (ρ_I_) or from a lower insulator-to-metal transition temperature (*T*_IMT_).^26^ Both parameters are affected by the number of excess carriers disrupting the electronic correlations, as in the case of high oxygen-vacancy concentration.^27,28^ In addition, geometrical factors and thermal conductivity also contribute. Thus, while the reduced *R*_I_ after forming directly links with a decreased ρ_I_, therefore *V*_TH_, the overall threshold reduction remains a combined contribution of several factors.

**Figure 4:**
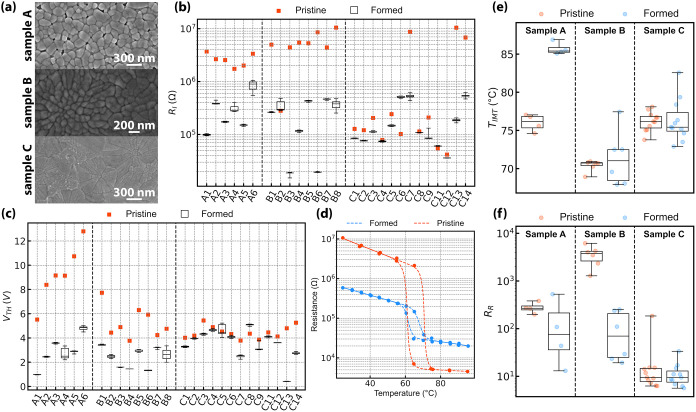
(a) SEM pictures of the sample morphology. (b, c) Extracted *R*_I_ and *V*_TH_ of pristine short-channel devices alongside a box-plot elaborated on the set of measurements after forming. *V*_TH_ in the pristine state is considered as the voltage value in which the first electroforming event occurs. (d) Example of variation of a short-channel device’s resistance (sample B) with the temperature before and after actuation of short-channel devices. (e, f) Box-plot of *R*_R_ and *T*_IMT_ before and after forming short-channel devices.

To evaluate the impact of electroforming on the thermal behavior of VO_2_, the temperature dependence of the resistance was measured before and after actuation for all of the samples. An example is reported in Figure 4d, emphasizing the effect of electroforming on both *T*_IMT_ and the resistance ratio (*R*_R_ = *R*_25 °C_/*R*_95 °C_) upon transition. Figure 4e summarizes the change in the *T*_IMT_ for several devices. With the exception of sample A showing a clear median increase of *T*_IMT_ (9.2 °C), samples B and C showcase a moderate median variation, with a larger spread of the temperature distributions compared to the initial sets and both positive and negative shifts observed in the *T*_IMT_ of the single devices. The direction of the shift is influenced by the differences in strain levels after forming, variations in microstructure, and local changes in phase composition.^29^ Sample A appears comparatively more robust to such factors owing to its higher homogeneity. Figure 4f reports the variation of the *R*_R_ for the same devices. Compared to the pristine condition, electroforming induces a decrease in the average *R*_R_, primarily associated with the reduced insulating resistance, while *R*_95 °C_ differs less from its original value.

A simplified model can be used to assess the *R*_R_ influence on the temperature spike (*T*_spk_) experienced by a device during actuation. In this framework, Joule heating is assumed to be the primary mechanism driving the transition, which occurs at the actuation temperature (*T*_TH_) when a conductive path is formed in the film under electrically triggered percolation avalanches.^21^ Because the VO_2_ switching time is significantly shorter (<subns to tens of ns)^30–32^ than the discharge time constant (>500 ns) of the parasitic capacitive elements, the device temporarily sustains the threshold voltage in its low-resistance phase (*R*_M_ = *R*_I_/*R*_R_), resulting in a rapid temperature increase. Assuming that cooling occurs through the substrate at temperature *T*_S_ via a thermal conductance G^th^, for an instantaneous transition with negligible latent heat contribution, *T*_spk_ can then be expressed as directly proportional to *R*_R_ (see Supporting Information for the full derivation and detailed discussions on assumptions)

1 $$T_{\mathrm{spk}}=T_{S}+R_{R}\times \left(T_{\mathrm{TH}}-T_{S}\right)$$

We infer that switching dynamics, latent heat, and thermal capacity of both the film and substrate reduce the effective peak temperature. Nevertheless, variations caused by spurious phase inclusions or grain-boundary resistances are expected to show only minor physical parameter fluctuations, negligible compared to the dominant effect of changes in *R*_R_. A similar conclusion applies to *T*_TH_, which may change with *T*_IMT_, but such variations are negligible relative to the orders-of-magnitude changes introduced by *R*_R_. The observed influence of *R*_R_ on the device SR reported in Table 1 supports the relevance of the model. Samples A and C, despite having similar thicknesses, exhibit very different survivability rates, which can be directly attributed to their differences in *R*_R_. In contrast, in sample B, the increased thickness provides more material to sustain connections after channel deformation, improving SR compared to sample A. Sample C, with its low *R*_R_, demonstrated devices that could operate with a minor to no degree of electroforming. Interestingly, samples that underwent natural electroforming (A and B), despite their large thickness and pristine *R*_R_ variations, appear to converge toward the same formed *R*_R_. The reduced *R*_R_ observed through all samples likely explains why formed devices ceased to undergo significant modifications. Finally, an analysis of the electrical characteristics of pristine devices was performed to understand the difference in device-to-device survivability rate within the same sample. However, no statistically significant correlations were found, emphasizing the influence of each channel’s specific morphology (shape of the grains, number of grain boundaries, voids, inclusions) on the final device survivability.

**Table 1:** Thickness of the Samples with the Relative Median *R*_R_ in Pristine and Formed States, the Median *R*_R_ Reduction, and the Survivability Rate

sample	A	B	C
Thickness	40 nm	100 nm	45 nm
*R*_R_ prist.	238	4280	18.2
*R*_R_ form.	28.8	26.4	9.4
Median reduction	–88%	–99%	–48%
Survivability rate	20%	40%	60%

We investigate the relation between microstructure and electroforming as a way to understand the formation of the conduction path. Structural elements exhibiting distinct behaviors during electroforming were identified through SEM inspection of the devices before and after actuation. Figure 5 illustrates how electroforming alters the channel morphology in two different sample C devices. Radial-like structures identified as nucleation points (NPs) during film deposition^22^ and phase inclusions appearing as small grain regions (SGRs) are visible prior to forming (left images). NPs act as structural weak points, more prone to alteration during electroforming, whereas SGRs tend to remain unaffected (right images). Postactuation, regions with elongated grains typically emerge, often accompanied by voids and hillocks.

**Figure 5:**
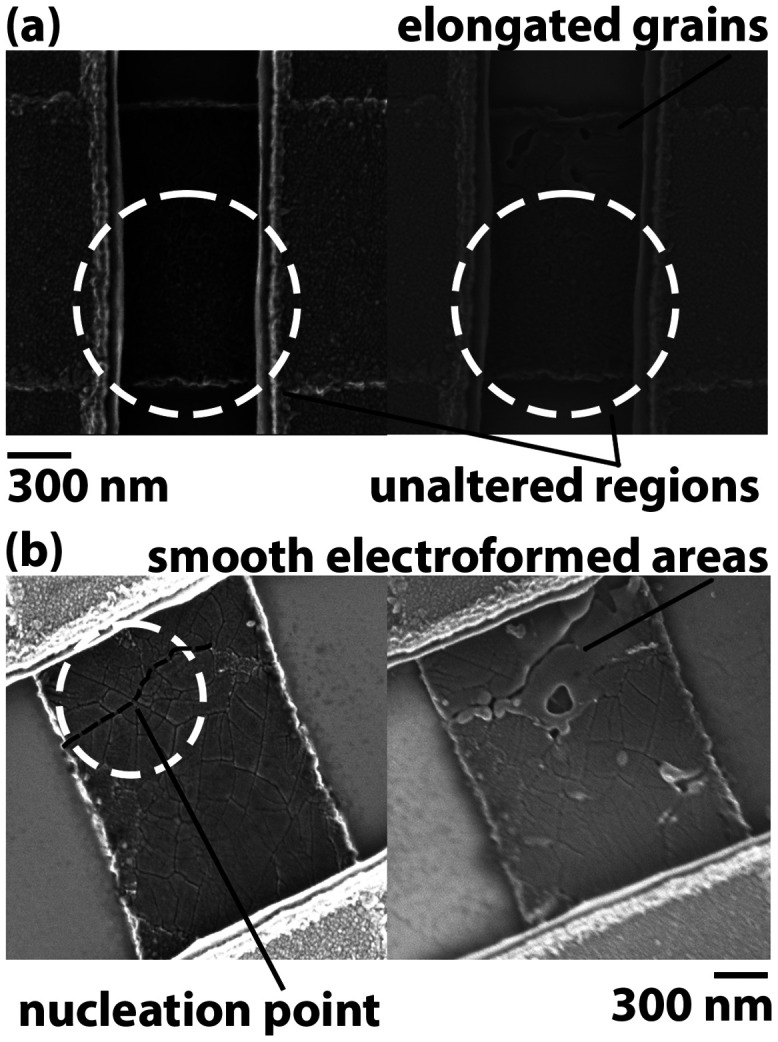
SEM pictures showing sample C short- and long-channel devices before and after electroforming. (a) SGR regions are usually untouched by the process, while the rest of the channel features elongated grains. (b) NP are observed to be more affected by the forming procedure, acting as a weak point in the device. Breakage often occurs at the long grain boundaries of NP.

To understand the relation between the microstructure and formation of the electroformed path, conductive AFM (C-AFM) maps were acquired with a moderate bias of 0.7–1 V. Figure 6 reports SEM, AFM topography, and C-AFM maps for two different long-channel pristine devices of sample C. The boundaries of the NP exhibit higher current following the radial shape (Figure 6a), while a lower conduction level is present in the SGR (Figure 6b). We speculate that the higher conduction along the boundaries of the NP relates to a higher concentration of oxygen vacancies^33^ locally favoring the IMT. Thus, this results in a higher probability of electroforming-driven structural changes in these regions. In Figure 7, SEM pictures of sample C long-channel pristine devices (“CL1” and “CL2”), with the related topography and current maps after electroforming, are shown. Device “CL1” is the same as that depicted in Figure 6b. After forming, the conductive left boundary of the NP (white dashed lines) cuts the channel into distinct areas, whereas the other boundary disappears as it is incorporated in a thick region resembling the smooth ones observed in the AFM experiment for sample A. The C-AFM reflects the morphology difference, as after forming, conduction is mostly carried by the area with more distinct grains rather than in the surrounding regions. In device “CL2”, the SGR (inside the dashed yellow lines) partially survives the forming process, whereas the rest of the channel presents a bulbous, irregular morphology. As for device “CL1”, points with the highest conduction are located at the interface between the heavily formed region and the SGR, avoiding the areas with a less clear grain structure.

**Figure 6:**
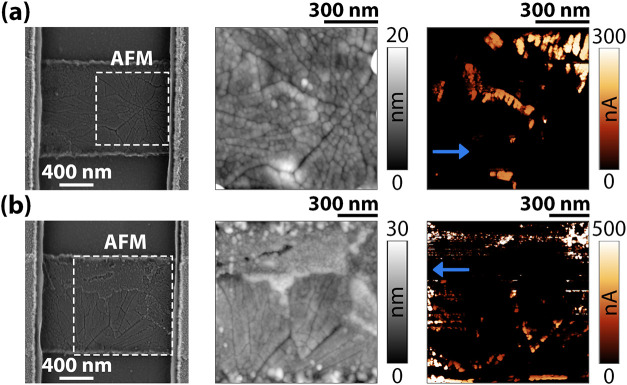
SEM and AFM/C-AFM pictures of two different sample C long-channel devices with a zoomed-in view of a NP (a) and a NP + SGR (b). The related C-AFM maps show several conductive points following the long grain boundaries of the NP, with poor conduction in the SGR. The blue arrows in the pictures refer to the direction of the ground contact for C-AFM (bias on the tip), introducing a small resistance gradient in the horizontal direction. The gradient does not, however, impact the vertical direction, which acts as a qualitative indication of the overall conduction.

**Figure 7:**
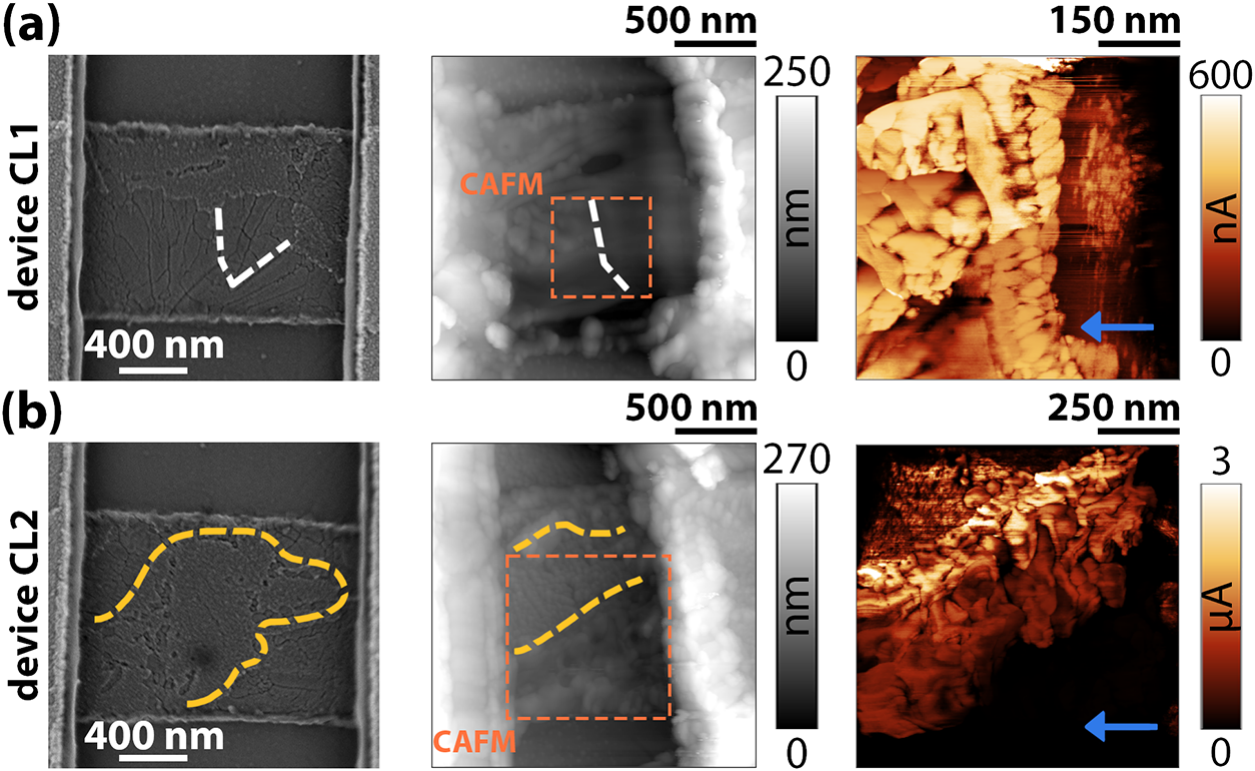
SEM, AFM topography, and C-AFM maps of two different sample C long-channel devices before (SEM pictures) and after electroforming (AFM/C-AFM). The forming was homogeneous for both devices, “CL1” and “CL2”, partially deforming the contacts, spreading material into the channel. The C-AFM maps, acquired in the specified area, underline the different conductivities of areas appearing with different morphologies as depicted by the topography map (elongated grains, smoother regions, SGR).

These observations highlight the key role of film microstructure and nonuniformities in the electroforming process, although their impact on device survivability rate is strictly dependent on both the resistance ratio *R*_R_ and the film thickness. Consequently, all of these elements must be accounted for when selecting device geometry, as longer channels statistically contain a greater number of weak regions and therefore ensure survivability only within a limited range of *R*_R_ and film thickness. In our experiments, long-channel devices could be successfully actuated only for sample C, consistent with its low *R*_R_.

### Phase Transformation and Electromigration

The rapid temperature increase at the IMT may be sufficient to induce a phase transformation, as endorsed by the presence of channel regions with different conduction properties. To gain deeper insight into microstructure and composition alteration after electroforming, TEM images for a pristine and formed device from sample B were acquired. Figure 8a,b reports the bright-field TEM (BF TEM) images for both kinds of devices, together with a SEM top view of the electroformed channel (inset in Figure 8b). The pristine device shows defined columnar grains, and a homogeneous VO_2_ M1 (*P*2_1_/*c*) phase is identified by selected area electron diffraction (SAED) analysis (Figure S2a). The BF TEM image of the electroformed device highlights low-density regions, hillocks, and voids in the channel, as well as the presence of grains with an irregular shape, whereas those beneath the contact remain unaffected. The minimal damage observed beneath the contact suggests that the transfer length is negligible compared with the channel dimensions, consistent with a relatively low contact resistance, implying a minor role of the contact in the process compared to the intrinsic film characteristics. This interpretation is further supported by measured *R*_95 °C_ values as low as 1 kΩ, which would lead to a more extended damaged region if the contact resistance were comparable. The SEM top view shows how the hillock extends in the entire width of the channel, with a clear separation with respect to the region where grains are still visible. Traces of phases with mixed oxidation state which can be indexed as V_3_O_5_ (*P*2/*c*) or V_3_O_7_ (*C*2/*c*) by SAED analysis (Figure S2c) were observed close to the hillock. The uncertainty in the crystal phase identification originates from the poor spatial resolution of the SAED technique, which led us to use scanning precession electron diffraction (SPED) experiments to record local diffraction patterns and resolve the individual crystal grains.^34^ The resulting pseudokinematical diffraction patterns were processed with ASTAR software for automatic crystal phase and orientation mapping.^35^ The crystal phase map of the pristine sample (Figure 8c) confirms the uniform VO_2_ M1 (*P*2_1_/*c*) phase, with a columnar shape and a strong out-of-plane texture as depicted in the orientation map in Figure 8e and confirmed by the {011} pole Figure (S3). Figure 8d illustrates the crystal phase map for the electroformed device, identifying the coexistence of V_3_O_5_ (*P*2/*c*) and V_2_O_3_ (R 3̅c), while discarding V_3_O_7_ (*C*2/*c*). The orientation map in Figure 8f highlights the transformation of the grain structure from columnar beneath the contacts to progressively stretched and completely elongated horizontally in the V_2_O_3_/V_3_O_5_ region.

**Figure 8:**
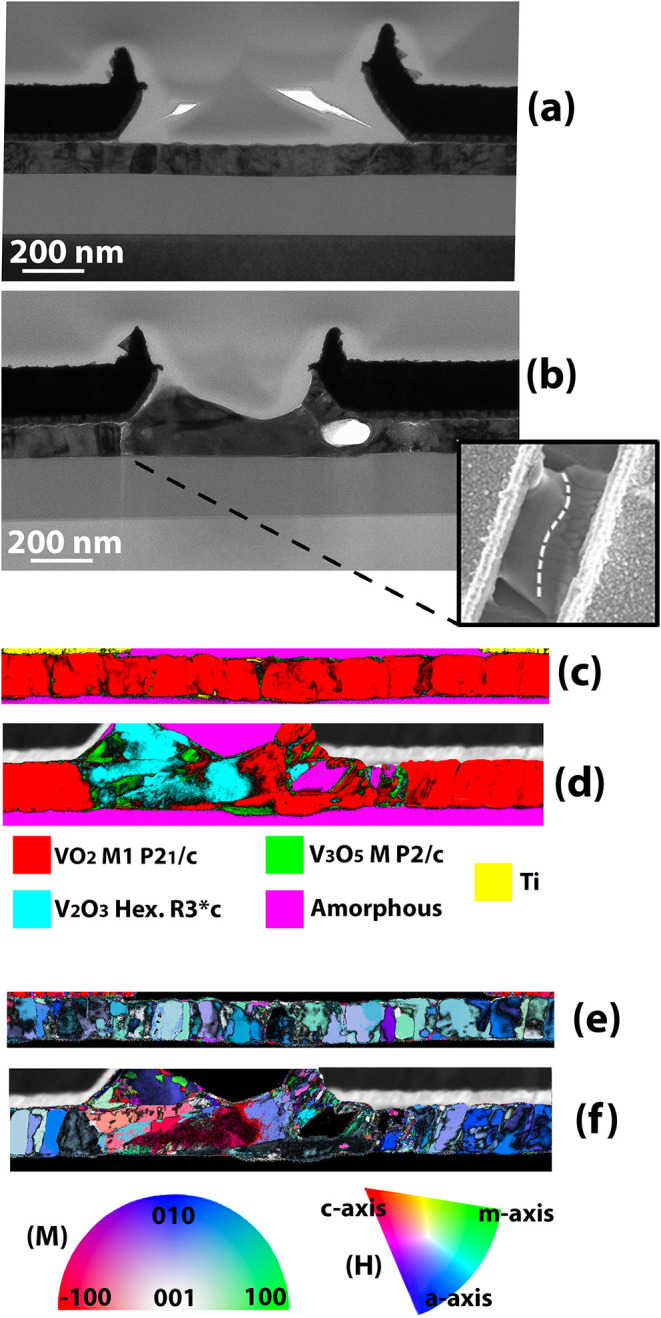
(a, b) BF TEM for a pristine (a) and electroformed (b) sample B device with an SEM inset of the top surface. Voids and hillocks are present after actuation, while below the contacts, the microstructures are unaltered. (c,d) SPED crystal phase maps of the devices underlining the transformation of VO_2_ M1 (*P*2_1_/*c*) in low-oxygen compounds after actuation. The phases are color-coded according to the legend. (e,f) Corresponding SPED crystal orientation maps, relative to the out-of-plane (vertical) direction. The color keys used to read the orientation of the monoclinic (M) (VO_2_, V_3_O_5_) and hexagonal (H) (V_2_O_3_) crystals should be used according to their spatial distribution, as identified in the related crystal phase maps. In the color maps, the contacts of the formed devices are overlapped with the grayscale BF image to guide the reading.

The presence of a compound with an inferior oxygen content agrees with previous observations in VO_*x*_-based devices.^16,17^ In particular, the channel geometry promotes oxygen release during electroforming, thereby favoring the formation of compounds with lower O/V ratios, such as V_2_O_3_ and V_3_O_5_. The change of electrical properties after actuation directly relates to the structural and composition changes in the channel. Particularly, we illustrate the consistent reduction of *R*_R_ as a the result of the phase mosaic after actuation by means of a finite element method (FEM) simulation. The film is discretized into 50 nm square grains, each of them existing either in a metallic or an insulating state, depending on their temperature in relation to the individual grain *T*_IMT_ and *T*_MIT_. The grains are connected to their neighbors via temperature-dependent resistances, representing their state; hence, the total film resistance is extracted as the overall resistance of the grain network. The quality of the FEM was assessed by matching the simulated thermal behavior of the resistance with the raw data from a pristine sample B device and a related analytical model (Figure 9b).

**Figure 9:**
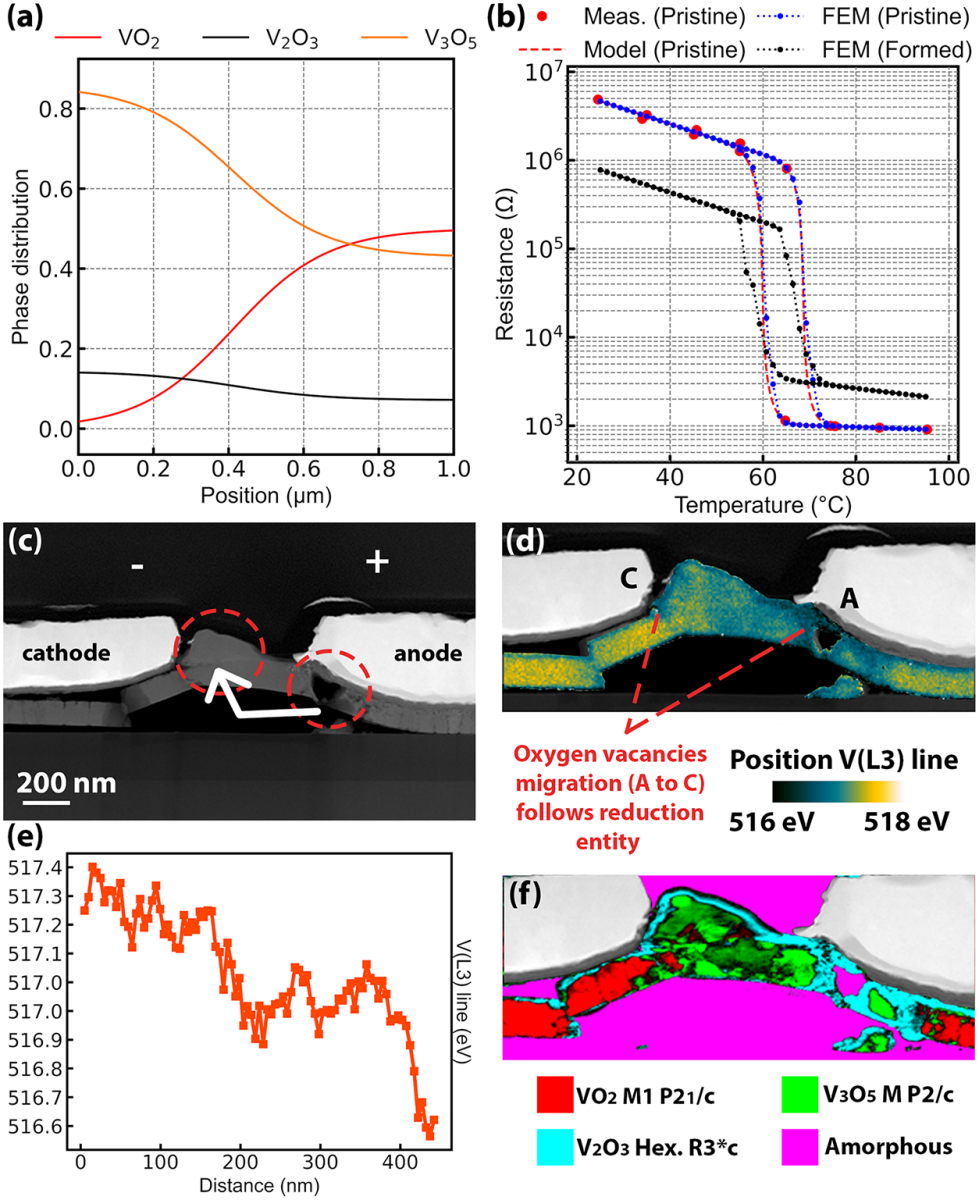
(a) Example of phase distribution in the channel. (b) Raw data, analytical model, and FEM simulation of the thermal behavior of a pristine device together with the FEM prediction of its electroformed state using (a) phase distribution. (c) HAADF STEM picture of an electroformed device. Low-density areas and a void are present in the anode proximity, while a hillock is formed at the cathode, indicating material transport. (d) EELS map showing the color-coded energy position of the V­(L_3_) line, with the HAADF STEM grayscale image of the contacts and substrate overlapped to guide the reading. Higher energy values, suggesting the presence of V^4+^, are observed in regions less damaged during the forming process. A progressive shift of the energy from 518 to 516 eV indicates the presence of V^3+^. (e) Plot of the energy shift of V­(L_3_) between the cathode (C) and anode (A) points shown in part (d). (f) SPED crystal phase map showing the phase composition in the channel. The HAADF STEM grayscale image of the contacts is overlapped to guide the reading.

Finally, FEM was used to emulate the phase mosaic provoked by the electroforming. V_2_O_3_ and V_3_O_5_ resistivity behavior were modeled using a similar approach, starting from reported data,^36,37^ and the channel thickness was corrected to emulate the material transport observed from the TEM lamellas. The chosen distribution of phases in the channel is reported in Figure 9a, and it was modeled based on experimental data provided by crystal phase maps measured by SPED, with a higher concentration of V_2_O_3_ and V_3_O_5_ close to one of the contacts, whereas the other is assumed to be more VO_2_-rich. Another scenario with a higher V_2_O_3_ fraction is provided in Figure S4. Figure 9b reports the resulting thermal behavior of the resistance, underlining the reduction of *R*_R_ as a consequence of both *R*_I_ reduction and an *R*_M_ increase. Overall, regardless of the dominant fraction being V_2_O_3_ or V_3_O_5_, both *R*_R_ and *R*_I_ are reduced, whereas the increase in *R*_M_ can be attributed either to an increased V_3_O_5_ phase fraction or a local geometry variation, such as bubble or void formation.

The formation of oxygen-deficient regions following electroforming relates to the increased presence of positively charged defects, such as oxygen vacancies. Figure 9c reports the high-angle annular dark-field (HAADF) STEM image of a formed sample B device together with the terminal polarity. The simultaneous presence of a void at the anode and a hillock at the cathode indicates electromigration in the channel, likely initiated by the enhanced defect diffusivity under the combination of high temperature and electric field during electroforming. Indeed, while the formation energy for oxygen vacancies in VO_2_ is reported to be 4.6 eV,^38^ the lowest energy barrier for oxygen-vacancy diffusion is estimated to be close to 0.55 eV.^39^ Figure 9d shows a map of the energy position of the V­(L_3_) fine structure line, measured by electron energy loss spectroscopy (EELS). An overall shift toward lower energy indicates a reduction in vanadium oxidation state from V^4+^ to V^3+^,^40,41^ a result corroborated by analyzing the energy position of the V­(L_2_) line and the V­(L_3_)/V­(L_2_) amplitude ratio (Figure S5). The energy position of V­(L_3_) exhibits a slight gradient from the anode to the cathode (Figure 9e), thus a higher oxidation state toward the cathode, which correlates with the gradient of material transport and with the possible direction of oxygen-vacancy migration, suggesting that they are primarily involved in the electromigration process. Moreover, the SPED crystal phase map on the same channel in Figure 9f shows that the gradient of V­(L_3_) energy position is present in a region having the same crystallographic phase (V_3_O_5_), enforcing a reduction operated by vacancies rather than the presence of a different phase. We highlight that a similar phenomenon also occurred upon AFM actuation of sample A, resulting in the formation of conical structures at the tip, acting as a cathode. Particularly, hillock formation in oxides biased by AFM tip is reported as a combination of electrothermal effects and defects, which influence the electrostatic interaction between the tip and the surface.^42,43^ We deduce that the occurrence of the electromigration in VO_2_ is strictly associated with the defects generated during electroforming, being independent of the film deposition method (ALD or PLD) or the contact material (Ti/Pt or Cr/Au), as well as the contact geometry that only affects the way the material thickens at the cathode following the electric field lines.

### Device Engineering by Current-Controlled Electroforming

Despite electroforming impacting device behavior, if the phenomenon is properly controlled, it can be exploited to enhance device survivability and to tune its switching characteristics. Indeed, an electrical preconditioning consisting of current-cycling (Figure S1) progressively reduces *R*_I_, artificially limiting *R*_R_, thus *T*_spk_. Considering sample B as a reference, the devices actuated following the preliminary preconditioning had a 40% survivability rate (Table 1), given the number of devices surviving both the preconditioning and the subsequent voltage cycling. Differently, a second group of devices tested in the absence of the current-cycling procedure resulted in a 0% survivability rate after the first voltage actuation, as the devices underwent breakdown during the IMT event, resulting in a spike-like feature of the current (Figure 10a). Particularly, the failure occurred for a median power value of 269 μW, which is comparable to the median power threshold (228 μW) required to actuate current-controlled electroformed devices, underlining the effect of a reduced *R*_R_. Figure 10b,c reports an example of *V*_TH_ and *I*_TH_ in 10^3^ voltage cycles for a device subjected to current preconditioning. The stochasticity is low, as indicated by a standard deviation of the *V*_TH_ distribution of 40.1 mV, corresponding to a coefficient of variation of 1.6%. Moreover, no degradation of the threshold values is observed during the cycling (see insets), suggesting high fatigue resistance in VO_2_ switches electroformed under controlled conditions.

**Figure 10:**
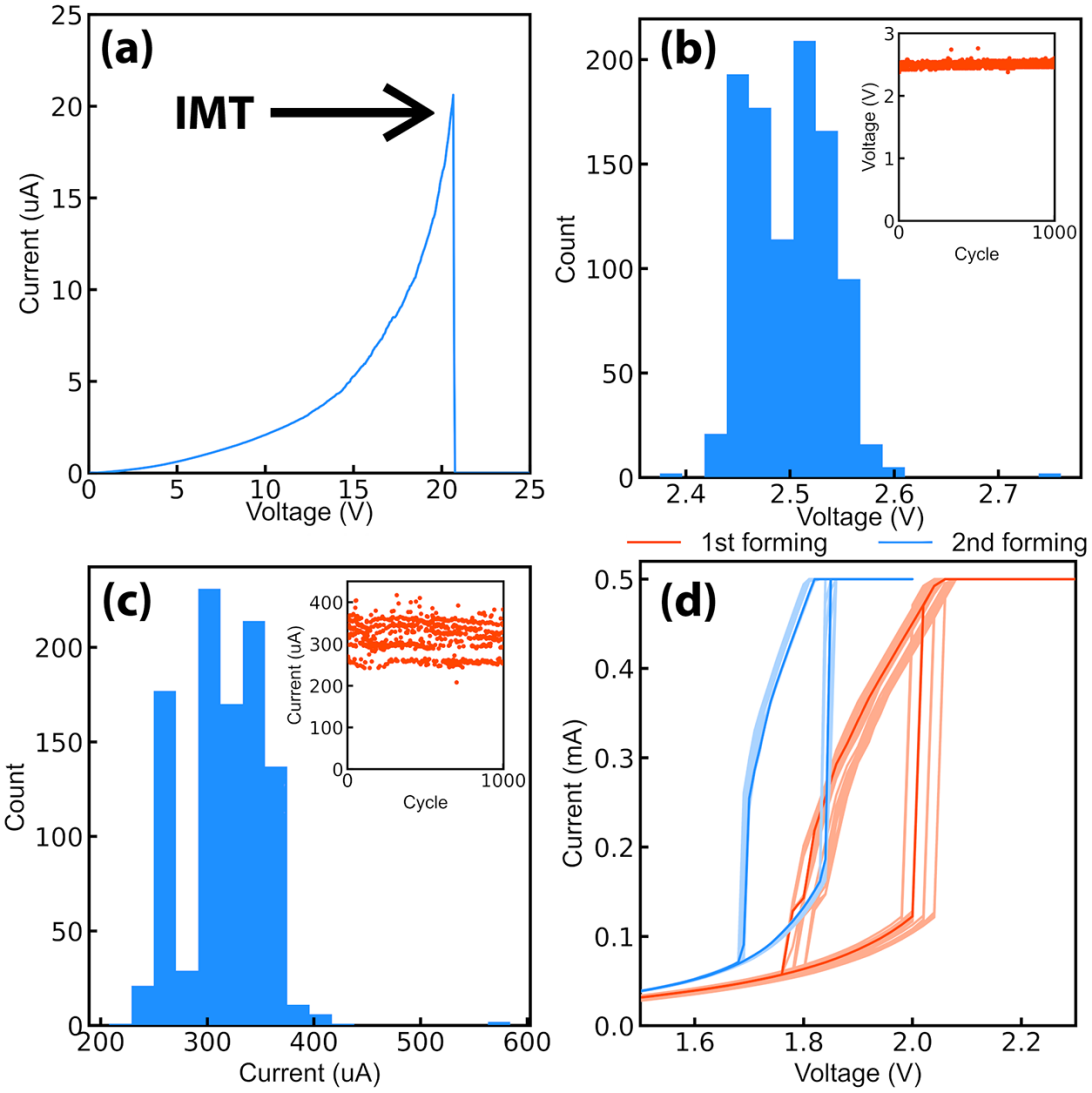
(a) Example of the *I*–*V* characteristic of a device not subjected to the preliminary current-cycling procedure. Failure is registered at the first IMT event. (b,c) *V*_TH_ and *I*_TH_ distributions in 10^3^ for a device subjected to controlled electroforming. The insets report the trend of the thresholds with the number of cycles, showing negligible variations. (d) Example of *I*–*V* characteristics tunability following a second current-controlled electroforming for the same device. The darker curves are the average *I*–*V* extracted in 100 consecutive voltage cycles, represented by a lighter color.

Current-controlled electroforming can be exploited as an additional degree of freedom to tune switching characteristics. Figure 10d reports the example of *I*–*V* characteristics for the same device undergoing current preconditioning twice. After the first electroforming event, the device already shows a high threshold stability in 100 voltage cycles. However, following the second current training, the stochasticity of *V*_TH_ and *I*_TH_ is further reduced, as the standard deviation of their distributions, respectively, decreases by 73.9% and 56.3%. Furthermore, the median power consumption is cut by 27% with a median of 146 μW required to trigger an IMT event. Importantly, the shape of the *I*–*V* characteristic is improved, now exhibiting the coexistence of vertical upward and downward transitions, which allows to exploit the device as an oscillating element in neuro-inspired circuits. Considered the origin of the change of the electrical characteristics mostly driven by oxygen depletion and phase transformation, the reconfiguration of the device proceeds unidirectional, requiring a higher current set point and resulting in a progressive *R*_I_ reduction (Figure S6).

## Conclusions

Electroforming in VO_2_ was described both at the nanoscale and in two-terminal devices, illustrating its primary effects on film morphology, electrical properties, and stochasticity. The phenomenon is intrinsic to polycrystalline VO_2_ films and is primarily caused by the high temperature spike at the insulator-to-metal transition, resulting in transport phenomena at the grain boundaries. The origin of the temperature spike was modeled as a function of the resistance ratio, showing how films with lower *R*_R_ values are more likely to survive the forming process or to be only marginally subjected to such alteration. Our experimental results reveal a practical limitation inherent to polycrystalline VO_2_ thin-film devices. Specifically, films that exhibit intrinsically high resistance ratios (*R*_R_) are prone to pronounced electroforming, a process that is highly stochastic and often accompanied by significant reliability risks. As a consequence, these initially high *R*_R_ values are not maintained under realistic operating conditions, but instead evolve toward lower effective *R*_R_ after forming. In contrast, devices intentionally engineered to exhibit lower intrinsic *R*_R_ can offer important advantages for applications that prioritize cycle-to-cycle reproducibility, narrow distributions of threshold voltage and current, and long-term endurance. This increased operational stability is achieved at the expense of reduced fan-out capability, highlighting a fundamental trade-off between ideal performance metrics and reliable device operation.

The work highlights how the impact of different deposition methods and parameters on the microstructure affects the way electroforming modifies the channel and thus the insulating resistance. In this regard, inspection of structural elements in the channel reveals the importance of a shorter length and a larger thickness to mitigate structural damages. C-AFM and SEM identify the grain boundaries of the nucleation points as the most likely structural elements to initiate the transition and undergo electroforming, leading to premature device failure. Moreover, the formation of low oxidation state compounds, such as V_3_O_5_ and V_2_O_3_, was confirmed by advanced TEM characterization, connecting the reduction of the upper threshold voltage and the resistance ratio upon actuation to the modification observed for both microstructure and phase composition, as further suggested by FEM simulation. The presence of electromigration in VO_2_ was identified and related to the mobility of defects created due to oxygen depletion. The EELS map showed how the combination of high electric field and temperature during electroforming results in a progressive change of vanadium oxidation state in the direction of material migration, evident by the simultaneity of voids at the anode and hillocks at the cathode.

Thus, this study clarifies the intrinsic nature of electroforming in polycrystalline VO_2_, highlighting the necessity of engineering film microstructure, device geometry, and appropriate training protocols to achieve reliable VO_2_ memristors.

## Methods

### Film Deposition

VO_2_ is deposited on a SiO_2_ (200 nm)/Si substrate using ALD and PLD techniques. Sample A (ALD) is 40 nm thick, and it is deposited starting from TEMAV (70 °C) and H_2_O precursors, and then it is annealed for 75 min at 450 °C under 75 mTorr oxygen pressure to crystallize it. Samples B (100 nm) and C (45 nm) are deposited from a V_2_O_5_ target by PLD, at 400 °C under an oxygen pressure of 6.6 mTorr with a subsequent anneal at 500 °C (sample B) and at 400 °C with an oxygen pressure of 7.5 mTorr (sample C).

### Sample Fabrication

The contacts of the sample characterized with AFM are 10 nm Cr and 100 nm Au deposited with an Alliance-Concept DP-650 sputtering machine. The pattern was realized with an MLA150 Maskless Aligner exploiting the LOR5A/AZ1512HS resist. The channel of the two-terminal devices was fabricated, patterning the surface with AZ1512HS resist and etching VO_2_ exploiting Cl_2_/Ar chemistry in an STS Multiplex ICP tool. Ti/Pt (20 nm/150 nm) contacts have been patterned by exploiting LOR5A/AZ1512HS resist and deposited with a Leybold Optics LAB 600H evaporator.

### Electrical Measurements

Electrical measurements have been performed with a Keithley 4200AS parameter analyzer at 25 °C when not utterly specified. Characteristics at the nanoscale have been acquired with the Asylum Research ES Cypher AFM system from Oxford Instruments using a Cypher Dual Gain ORCA Holder (*I*_max_ = 10 μA) for current maps and a high voltage externally controlled by a Keithley 4200AS (*I*_max_ variable) for locally forming the surface. The environmental control system (ES) allowed for controlling the temperature and gas environment during the measurements. All of the samples have been dehydrated for 2 min at 100–120 °C under N_2_ flux and subsequently kept close to 30 °C during investigation. B-doped diamond tips from ADAMA Innovations and Ti/Ir-coated Si tips from Oxford Instruments were used.

### Transmission Electron Microscopy

Cross-sectional samples were prepared for TEM analysis by a Ga focused ion beam (FIB), using a Zeiss NVision 40 instrument. A Thermo Fischer Scientific Talos F200S instrument was used for bright-field (BF) imaging and selected area electron diffraction (SAED). It was operated at 200 kV and used a Ceta CMOS camera. An aberration-corrected Thermo Fisher Scientific Titan Themis^3^ microscope operated at 300 kV was used for high-angle annular dark-field (HAADF) STEM, scanning precession electron diffraction (SPED), and electron energy loss spectroscopy (EELS). The SPED data were acquired with a NanoMEGAS Topspin system coupled with a Quantum Detectors MerlinEM direct detection camera in nanobeam configuration. A precession angle of 1° and a convergence angle of 745 μrad, corresponding to a diffraction-limited spatial resolution of 1.7 nm, were used. The SPED data were processed with the NanoMEGAS ASTAR software suite to generate the crystal phase and orientation maps.^35^ The pattern-matching analysis was carried out by considering the following VOx crystal phase candidates: VO_2_ (*P*2_1_/*c*), V_2_O_3_ (R 3̅c), V_2_O_5_ (*Pmmn*), V_3_O_5_ (*P*2/*c*), V_3_O_7_ (*C*2/*c*), and V_5_O_9_ (P1̅). The EELS data were captured with a Gatan GIF Continuum K3 spectrometer. The dual-EELS data were processed using GMS software, where the L_2_ and L_3_ fine structure lines of vanadium were fitted with Gaussians to extract their peak position and maximum amplitude, after taking care to compensate for the slight 0-loss energy shift across the whole map.

## Supplementary Material



## Notes

Detailed thermal model derivation, additional experimental procedures and data, additional computation data including low-oxygen content compounds (PDF)
