# Supplementary material for: Electroforming in VO2 Switch: Phase Transformation and Electromigration Phenomena
Source: ACS Nano. 2026 Apr 1;20(14):10962–73. doi: 10.1021/acsnano.5c19805 (PMC13085912; doi:10.1021/acsnano.5c19805)
Supplement: Supplementary file 1 [file nn5c19805_si_002.pdf]

Supporting Information

Electroforming in VO_2 Switch: Phase Transformation and Electromigration Phenomena

Vanessa Conti*, Cyrille Masserey*, Victor Boureau,
Anna Varini, Igor Stolichnov, and Adrian Mihai Ionescu

1 Appendix (A1)

1.1 Model derivation

A simplified model was developed to estimate the theoretical maximum temperature spike (T_{spk}) reached by the device at the moment of transition. The model does not aim to provide absolute temperature values but it rather illustrates the influence of device and film parameters on the transient thermal behavior. The device can exist in two states: a conductive (metallic) state with resistance R_M, and an insulating state with resistance R_I. The device switches to the metallic state when its temperature (T_D) exceeds the threshold temperature (T_{TH}), and returns to the insulating state when T_D falls below the hold temperature (T_{hld}).

The Joule heating–driven percolation mechanism governing VO_2 memristor switching ensures that the electrically driven threshold temperature T_{TH} is distinct from, and typically lower than, the intrinsic insulator-to-metal transition temperature (T_{IMT}). A similar relationship applies between T_{hld} and the metal-to-insulator transition temperature (T_{MIT}). [1]. The two resistive states are related through the resistance ratio (R_R), defined as:

$$R_R = \frac{R_I}{R_M} \tag{S1}$$

The electrical power (P^{el}) dissipated through the device is the only source of heating, and the thermal dissipation occurs through a thermal conductance (G^{th}) connecting the device to the substrate, which acts as a heat sink at temperature T_S. Device geometry has a strong influence on G^{th}; however, the thermal conductivity of VO_2 is similar in both its insulating and metallic phases [2]. Therefore, the thermal conductance is considered to remain constant throughout the phase transition.

Thus, the steady-state temperature of the device can be expressed independently of its state using Fourier's law of thermal conduction:

$$P^{el} G^{th} = T_D - T_S \tag{S2}$$

The voltage threshold (V_{TH}) and current threshold (I_{TH}) correspond to the voltage and the current required to dissipate enough electrical power

to achieve a transition:

$$\begin{aligned} V_{TH} &= \sqrt{(T_{TH} - T_S)G^{th}R_R R_M} \\ I_{TH} &= \sqrt{(T_{TH} - T_S)\frac{G^{th}}{R_R R_M}} \end{aligned} \tag{S3}$$

Assuming the transition occurs instantaneously relative to other effects, such as the discharge of parasitic capacitors (C) elements, the threshold voltage is briefly maintained while the device is already in its low-resistance metallic state. This results in a transient electrical power spike (P^{el}_{spk}):

$$P^{el}_{spk} = \frac{V_{TH}^2}{R_M} = (T_{TH} - T_S)G^{th}R_R \tag{S4}$$

In this case, the peak temperature rise of the device can be derived directly from the thermal conduction equation (S2) presented earlier:

$$T_{spk} = T_S + R_R \times (T_{TH} - T_S) \tag{S5}$$

The expression shows no explicit dependence on the thermal conductance, implying a theoretically geometry-independent temperature spike.

1.2 Instantaneous device transition

In this model the device transition is considered instantaneous. To support the validity of this assumption, we estimated the parasitic capacitance of our measurement setup to be approximately 200 pF. Using the median metallic-state resistance values, the corresponding RC discharge time constants

were estimated to be 3.08 μs, 524 ns, and 2.12 μs for samples A, B, and C, respectively. In comparison, phase transition times reported in the literature [3] range from approximately 300 fs down to sub-femtosecond timescales, i.e., at least six orders of magnitude shorter. These estimates are derived from various pump–probe techniques. Other studies report longer electrically driven switching times attributed to Joule-heating-induced thermal dynamics. Particularly, reported simulated [4] and experimentally measured [5, 6] Joule-heating driven switching times of VO_2 devices at room temperature under abrupt voltage pulses indicate upper bounds on the order of 10 ns, still more than 50 times shorter than the fastest RC discharge time constant estimated in our measurements. Importantly, these reported switching times predominantly reflect thermal conduction and the heat capacity of the VO_2 film and substrate required to raise the device temperature to the transition threshold. This interpretation is supported by simulation studies that accurately reproduce switching times without explicitly accounting for filamentary conduction or latent heat effects [4]. While such studies are highly relevant for understanding the maximum achievable operating frequency of VO_2 based memristors, they do not directly capture the intrinsic phase transition and filament formation timescale, which is treated as instantaneous in our model. Instead, they primarily describe thermal ramp-up dynamics from ambient conditions, whereas in our framework the film is assumed to be already at, or very close to, the transition temperature.

This assumption therefore defines the validity domain of the present model.

If the parasitic discharge time constant τ_{dis} was close to the intrinsic device transition timescale, the effective temperature spike would be substantially reduced. This consideration suggests two primary strategies for improving device survivability: minimizing the metallic-state resistance and reducing parasitic capacitances in the measurement and circuit environment.

1.3 Negligible latent heat

To assess the validity of the negligible latent heat assumption, the energy required to induce a phase transition across the entire device can be estimated and compared with the voltage drop across the parasitic capacitance necessary to supply this energy.

For this estimation, we assume that the entire VO_2 film is already at the transition temperature, such that only the latent heat associated with the phase transition must be supplied. The device film volume is first estimated and multiplied by the density of VO_2 in the M1 phase (4340 kg·m^{-3}, as reported in the literature [7]) to obtain the film mass. This mass is then multiplied by the reported latent heat of the VO_2 M1–R transition (4184 J·mol^{-1} [8]) to estimate the total switching energy. Using this approach, the energies required for samples A, B, and C are estimated to be 8.76 pJ, 21.9 pJ, and 9.85 pJ, respectively. The corresponding voltage drops across the parasitic capacitance (assumed to be 200 pF) needed to supply these energies are approximately 87 mV, 893 mV, and 98.5 mV. These voltage drops correspond to approximately 0.97%, 4.38%, and 2.46% of

the pristine threshold voltage for samples A, B, and C, respectively. Notably, this estimation assumes that the entire channel undergoes the phase transition simultaneously. In practice, the electrically driven transition proceeds through a percolative regime in which only a small fraction of the film volume initiates the transition. Consequently, this analysis represents an upper bound on the impact of latent heat and further supports the conclusion that latent heat effects have a limited influence on the validity of the present model.

2 Supplementary Figures

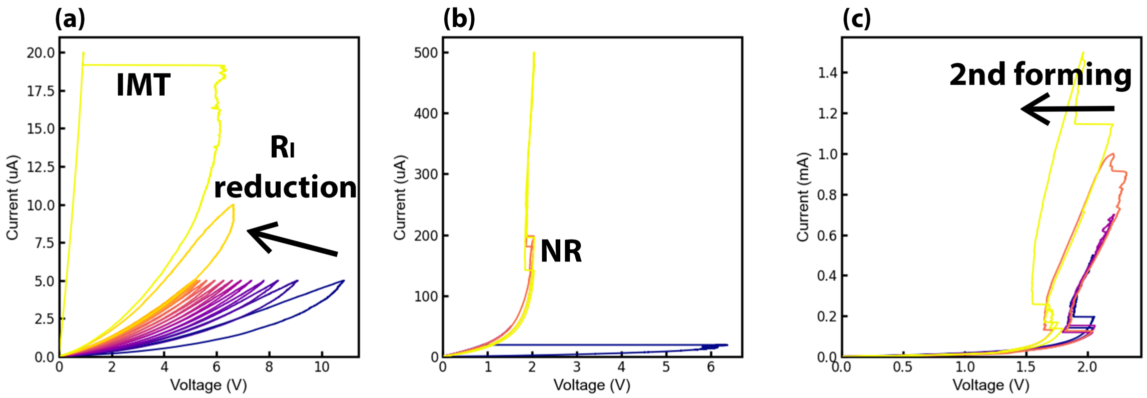

Figure S1: Example of the preliminary current-preconditioning to induce controlled electroforming. Current step in the sweep is chosen to be three-order of magnitude less than the maximum current value. As the current is applied, the voltage increases showing a hysteretic behavior during the backward sweep, and noisy-features are observed as a consequence of the progressive R_I (R_R) reduction. The channel is considered subjected to homogeneous electroforming as soon as a dramatic snap-back of the voltage appears in the V-I characteristics for a critical current value I_c. This coincides with the first IMT event. Often, no negative-resistance region (NR) appears following the IMT. Current higher than I_c is applied to the device up to a voltage-regulated region, followed by the appearance of the NR. As soon as the NR is present, the device can be tested with a voltage sweep. (a) Progressive current sweeps until the IMT event. The current range is increased only when the hysteresis closes. (b) Appearance of the NR following the situation depicted in (a) plot. (c) Example of a second forming leading to a change in the NR region.

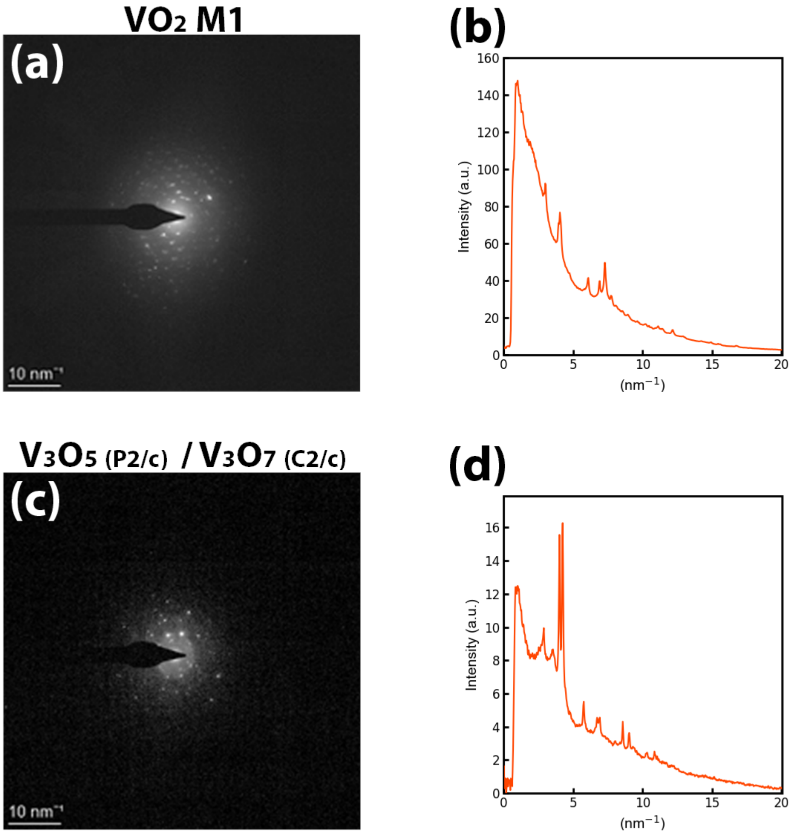

Figure S2: Selected area electron diffraction (SAED) patterns. (a) SAED of a pristine sample B device, enclosing several grains, for which the azimuthlal integration of the Bragg scattering intensities displayed in (b) is matching with VO_2 M1. (c) SAED of a formed sample B device, enclosing several grains. The indexation of the azimuthal integration of the Bragg scattering intensities shown in (d)

is uncertain for this pattern, with V_3O_5 or V_3O_7 as possible candidates.

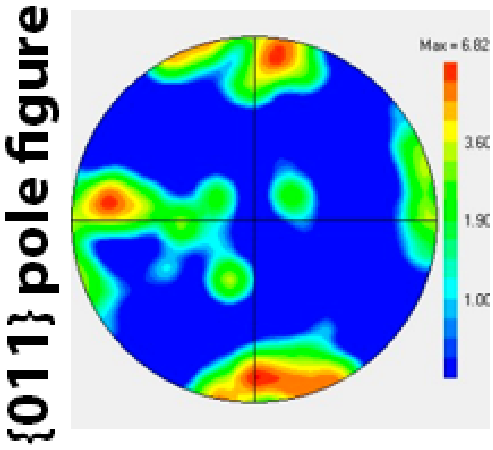

Figure S3: {011} pole figure obtained from the orientation map of a pristine sample B device. The channel exhibits a strong texture in which a majority of the {011} planes are oriented out-of-plane, relative to the sample substrate.

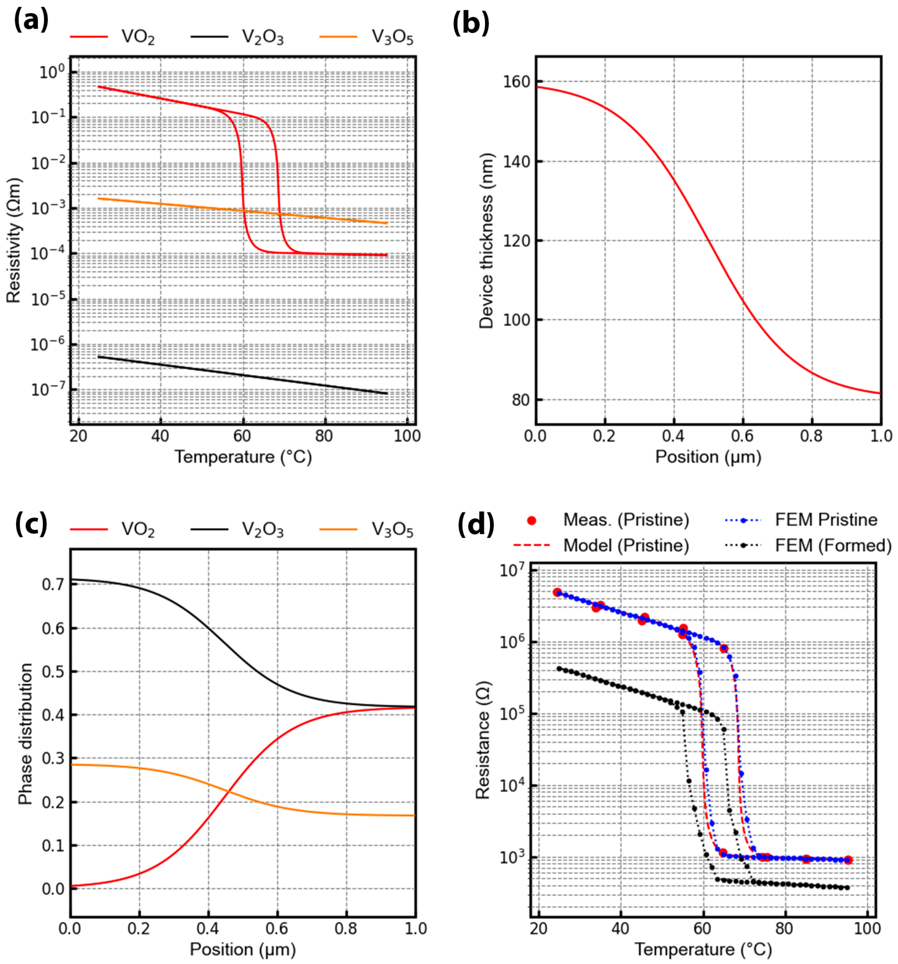

Figure S4: (a) Resistivity vs Temperature curves used for VO_2, V_2O_3 and V_3O_5 in the investigated temperature range. (b) Thickness correction to compensate material transport in the channel. (c) Alternative scenario for the phase distribution with V_2O_3 as dominant lower-oxygen compound. (d) Resulting FEM simulation of the Resistivity vs Temperature curves for a device with the phase distribution depicted in (c).

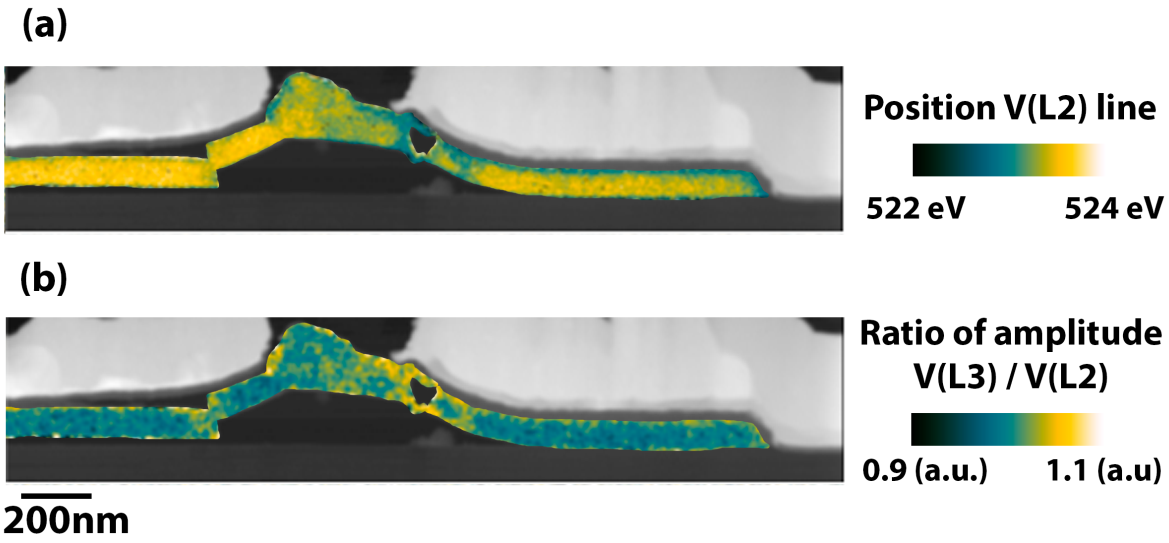

Figure S5: Electron energy loss spectroscopy (EELS) maps of the same formed sample B device showing the energy position of the $V(L_2)$ fine structure line (a) and the $V(L_3)/V(L_2)$ amplitude ratio (b). A shift towards lower energy of $V(L_2)$ or an increase of $V(L_3)/V(L_2)$ amplitude ratio suggests a reduction in the V oxidation state [9, 10]. The EELS signal (color-scale map) is overlapped to the greyscale HAADF image.

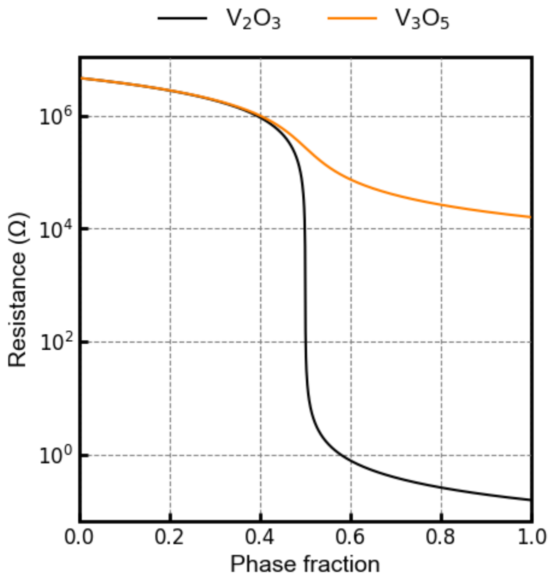

Figure S6: R_I trend with increasing percentage of V_2O_3 and V_3O_5. R_I behavior is analytically calculated assuming an homogeneous distribution of the low-oxidation state compound in the channel. The steep drop is associated to the formation of a preferred conductive path through V_2O_3 or V_3O_5 when the content of this compound becomes comparable with the one of VO_2.

Supporting Information References

(1) Kumar, S.; Pickett, M. D.; Strachan, J. P.; Gibson, G.; Nishi, Y.; Williams, R. S. *Advanced Materials* **2013**, *25*, 6128–6132, DOI: 10.1002/adma.201302046.

(2) Kizuka, H.; Saida, T.; Inoue, T.; Funakubo, H.; Yamaguchi, T.; Sugimoto, H.; Nakae, M.; Shimizu, S. *Japanese Journal of Applied Physics* **2015**, *54*, 053201, DOI: 10.7567/JJAP.54.053201.

(3) Yang, Z.; Ko, C.; Ramanathan, S. *Annual Review of Materials Research* **2011**, *41*, 337–367, DOI: 10.1146/annurev-matsci-062910-100347.

(4) Zhang, Y.; Ramanathan, S. *Solid-State Electronics* **2011**, *62*, 161–164, DOI: https://doi.org/10.1016/j.sse.2011.04.003.

(5) Stefanovich, G.; Pergament, A.; Stefanovich, D. *Journal of Physics: Condensed Matter* **2000**, *12*, 8837, DOI: 10.1088/0953-8984/12/41/310.

(6) Chae, B.-G.; Kim, H.-T.; Youn, D.-H.; Kang, K.-Y. *Physica B: Condensed Matter* **2005**, *369*, 76–80, DOI: https://doi.org/10.1016/j.physb.2005.07.032.

(7) Stefanovich, G.; Pergament, A.; Stefanovich, D. *Journal of Physics: Condensed Matter* **2000**, *12*, 8837, DOI: 10.1088/0953-8984/12/41/310.

(8) Cao, J.; Ertekin, E.; Srinivasan, V.; Fan, W.; Huang, S.; Zheng, H.; Yim, J. W. L.; Khanal, D. R.; Ogletree, D. F.; Grossman, J. C.; Wu, J. *Nature Nanotechnology* **2009**, *4*, 732–737, DOI: 10.1038/nnano.2009.266.

(9) Laffont, L.; Wu, M.; Chevallier, F.; Poizot, P.; Morcrette, M.; Tarascon, J. *Micron* **2006**, *37*, Proceedings of the International Workshop on Enhanced Data Generated with Electrons (EDGE), 459–464, DOI: https://doi.org/10.1016/j.micron.2005.11.007.

(10) Park, K.; Cho, J.; Lee, S.; Cho, J.; Ha, J.-H.; Jung, J.; Kim, D.; Choi, W.-C.; Hong, J.-I.; You, C.-Y. *Advanced Functional Materials*, *n/a*, 2422966, DOI: https://doi.org/10.1002/adfm.202422966.
